# Supplementary figures and images for: Occurrence of Giardia duodenalis in Cats from Queretaro and the Risk to Public Health
Source: Animals (Basel). 2023 Mar 20;13(6):1098. doi: 10.3390/ani13061098 (PMC10044519; doi:10.3390/ani13061098)

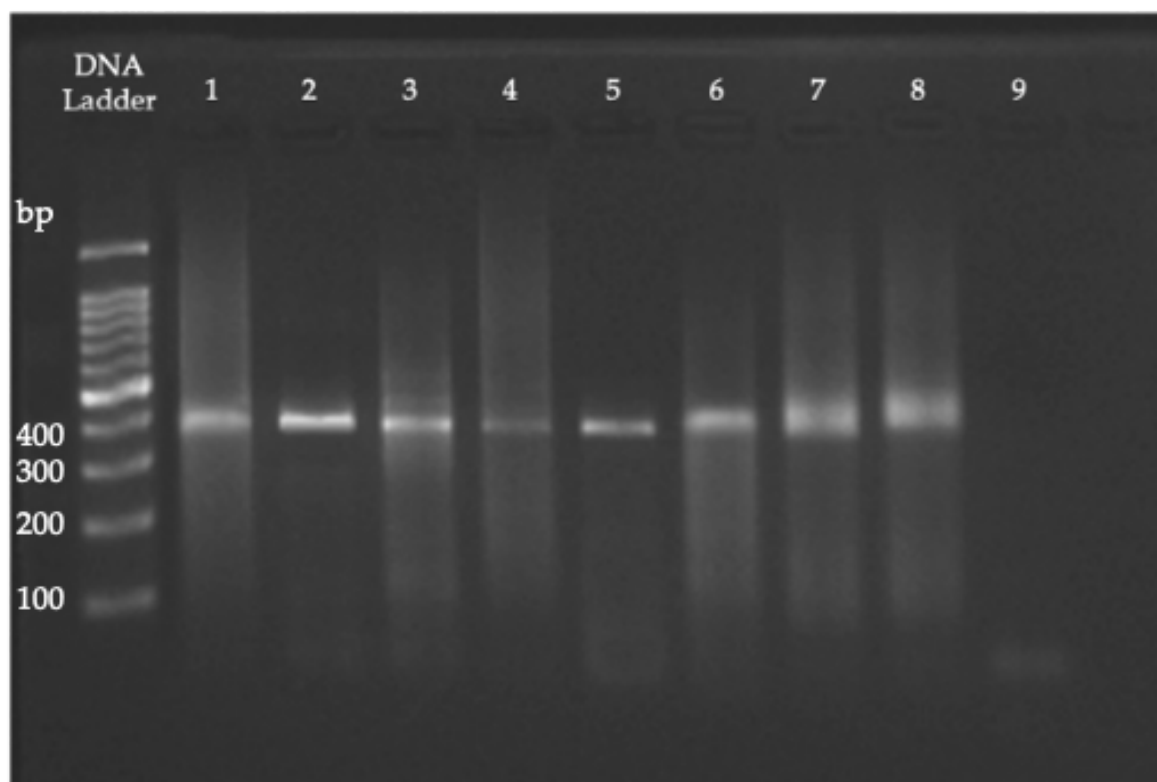

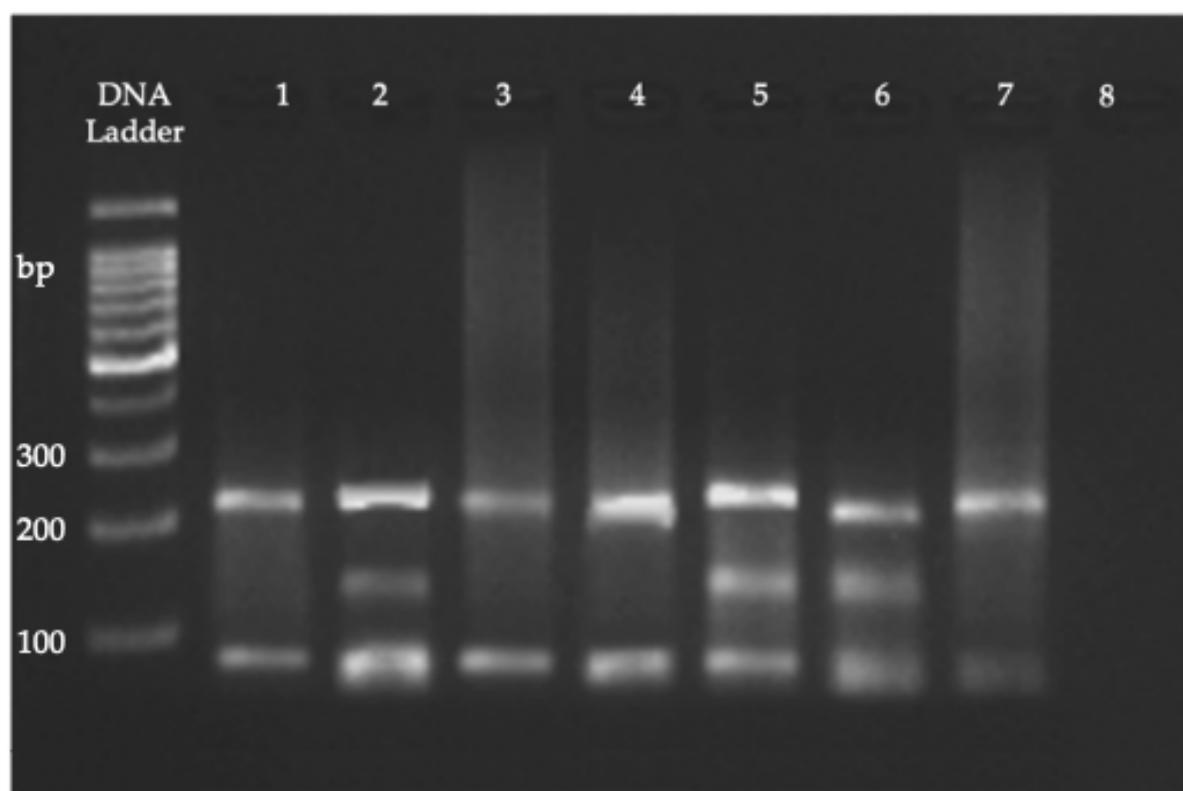

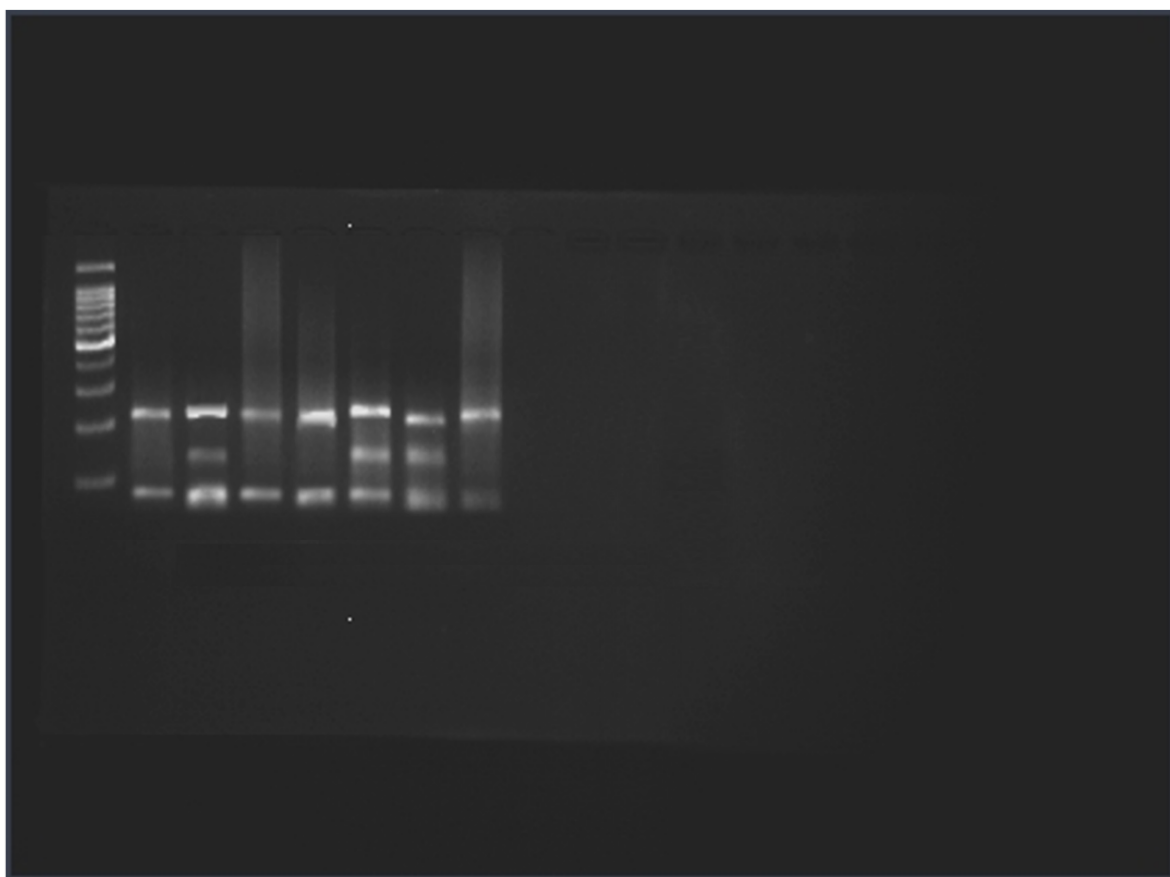

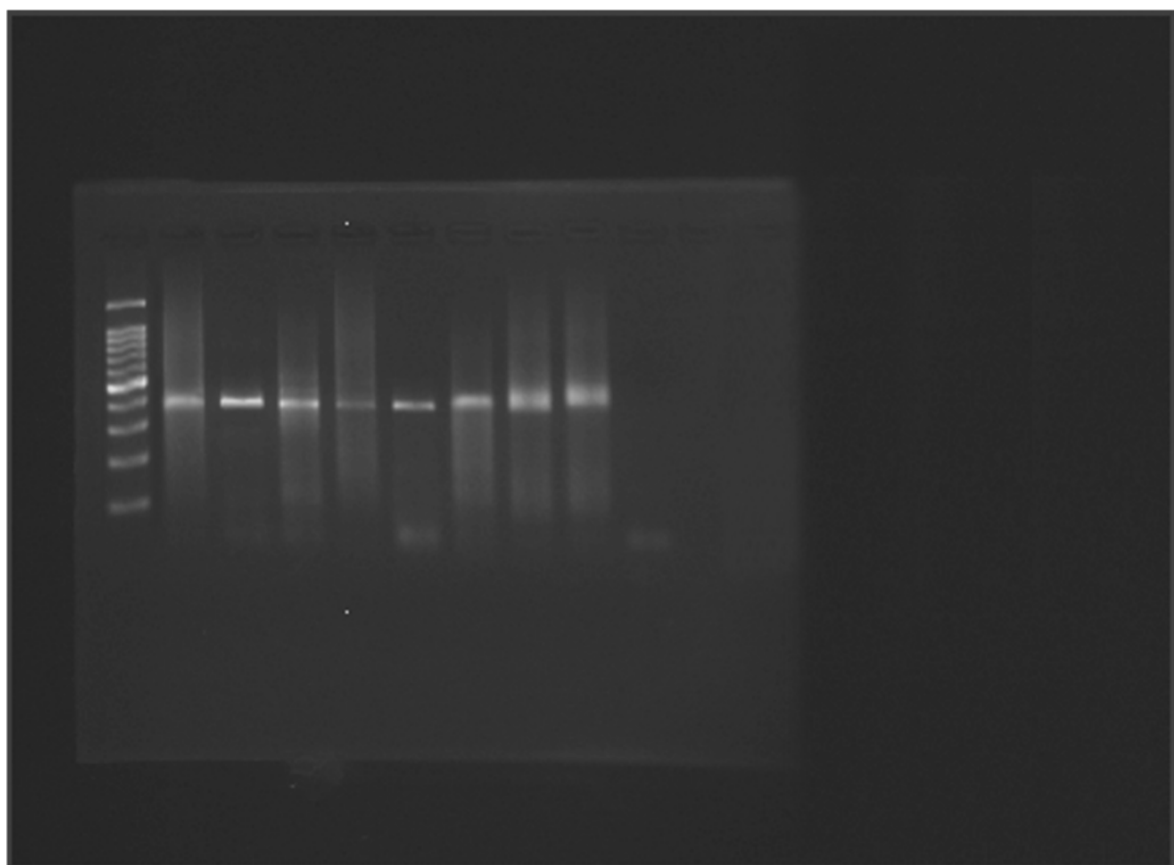

Supplement: Supplementary file 1 [file animals-13-01098-s001.zip › animals-2223531-supplementary.pdf]
